# Lactulose use among patients with alcohol-related liver cirrhosis as a surrogate marker of hepatic encephalopathy: prevalence and association with mortality - a Danish nationwide cohort study

**DOI:** 10.1007/s11011-025-01533-w

**Published:** 2025-01-18

**Authors:** Emma Celia Herting, Morten Daniel Jensen, Peter Jepsen

**Affiliations:** 1 Department of Hepatology and Gastroenterology, Aarhus University Hospital, Aarhus N, Denmark; 2 Institute of Clinical Medicine, Aarhus University, Aarhus, Denmark; 3 Department of Clinical Epidemiology, Aarhus University Hospital, Aarhus, Denmark; 4 Department of Hepatology and Gastroenterology, Aarhus University Hospital, Palle Juul-Jensens Boulevard 99, C116, DK-8200 Aarhus N, Denmark

**Keywords:** Alcohol-related liver disease, Lactulose, Hepatic Encephalopathy, Mortality, Surrogate marker, Prevalence

## Abstract

*Background & aims*: Hepatic encephalopathy (HE), one of the most serious prognostic factors for mortality in alcohol-related cirrhosis (ALD cirrhosis), is not recorded in Danish healthcare registries. However, treatment of HE with lactulose, the universal first-line treatment, can be identified through data on filled prescriptions. This study aimed to investigate if lactulose can be used as a surrogate marker of HE. *Methods*: We used Danish healthcare registries to establish a cohort of patients with ALD cirrhosis diagnosed in 2000–2018. Lactulose users were identified using data on filled prescriptions. We computed the prevalence and cumulative incidence of lactulose use, and then matched lactulose users with cohort members who were not using lactulose. This matched dataset was used to identify predictors of lactulose initiation, and to examine the association between lactulose use and all-cause mortality. *Results*: We included 23,089 patients, among whom we identified 4,789 first-time lactulose users. The prevalence of lactulose usage rose to 11% within the first 6 months after ALD diagnosis and reached 19% 5 years after inclusion. The 1-year cumulative incidence of lactulose use was 31%, and predictors of initiating lactulose use were history of severe liver disease (ascites, portal hypertension, spontaneous bacterial peritonitis or gastrointestinal bleeding) or hepatocellular carcinoma. Lactulose use was associated with higher mortality (adjusted hazard ratio 1.61 [95% confidence interval 1.53; 1.69]). *Conclusions*: Lactulose is used by 10–20% of patients with ALD cirrhosis, primarily those with severe cirrhosis, and lactulose users have a markedly higher mortality than nonusers. We conclude that lactulose use can be used to estimate the prevalence of HE and to reduce confounding from HE, but it should not be used as a surrogate for HE in studies with HE as an outcome.

**Supplementary Information:**

The online version contains supplementary material available at 10.1007/s11011-025-01533-w.

## Introduction

Hepatic encephalopathy (HE) is primarily seen in patients with cirrhosis and associated with a marked increase in mortality (Jepsen et al. 2010; Thursz et al. 2018; Häussinger et al. 2022). Hyperammonemia and systemic inflammation are important parts of the pathogenesis of HE (Thomsen et al. 2024), and the clinical aspects are characterized by personality changes, intellectual impairment, and decreasing level of consciousness (Shawcross et al. 2016). Therefore, information on HE is important for many studies of patients with cirrhosis, but unfortunately, there is no diagnosis code for HE in the 10th edition of the International Classification of Diseases (ICD-10). The first-line treatment of HE is lactulose (Vilstrup et al. 2014; Poordad 2015), and a United States study found that prescriptions for lactulose identified overt HE with a sensitivity of 0.77 to 0.95 and a specificity of 0.95 to 0.98 in two separate cohorts (Tapper et al. 2021). Therefore, the study concluded that lactulose prescriptions might be used as a surrogate for HE, at least in some study types. For instance, the prevalence of lactulose use could be used to estimate HE prevalence, e.g., for sample size estimations, and controlling for lactulose use might minimize confounding due to HE.

Given this background, we first examined the prevalence of lactulose use among Danish patients with alcohol-related cirrhosis (ALD cirrhosis). Second, we examined the incidence and predictors of initiating lactulose treatment, hypothesizing that presence of other cirrhosis complications predict lactulose treatment. Third, we examined the association between lactulose use and mortality among Danish patients with ALD cirrhosis, recognizing that a higher mortality among lactulose users could be considered as validation for lactulose use a surrogate of HE(Jepsen et al. 2010).

## Materials and methods

### Patient selection

This study was based on data from Danish healthcare registries from 1 January 1995 till 1 January 2019. We used the National Patient Registry, which covers all Danish hospitals and contains data on all in- and outpatient contacts since 1977, and the National Presciption Registry, which contains data on all filled prescriptions at community pharmacies in Denmark since 1994 (Pottegård et al. 2017).

We used the National Patient Registry to establish a cohort of patients ≥ 18 years of age with a first-time diagnosis of ALD cirrhosis between 1 January 2000 and 1 January 2019; all diagnoses were identified by ICD-10 codes (Schmidt et al. 2015). Patients with a diagnosis of ALD cirrhosis between 1995 and 1999 were excluded. Patients entered the cohort on their ‘ALD date‘, which was defined as either the earliest discharge date of a hospital admission or the date of the first outpatient visit coded with an ALD cirrhosis diagnosis code (whichever came first). Patients were excluded if they had filled a prescription for lactulose before the ALD date. Usage of lactulose after the ALD date was identified using data from the National Presciption Registry. Of note, lactulose can be purchased over-the-counter, and we could not identify such purchases. Lactulose is a low-cost drug, with prices at about 20 euros per liter and for some patients, it is possible to have a subsidy which further decreases the cost (Kaplan 2021).

### Study design

#### Prevalence and cumulative incidence of lactulose use

**Fig. 1:**
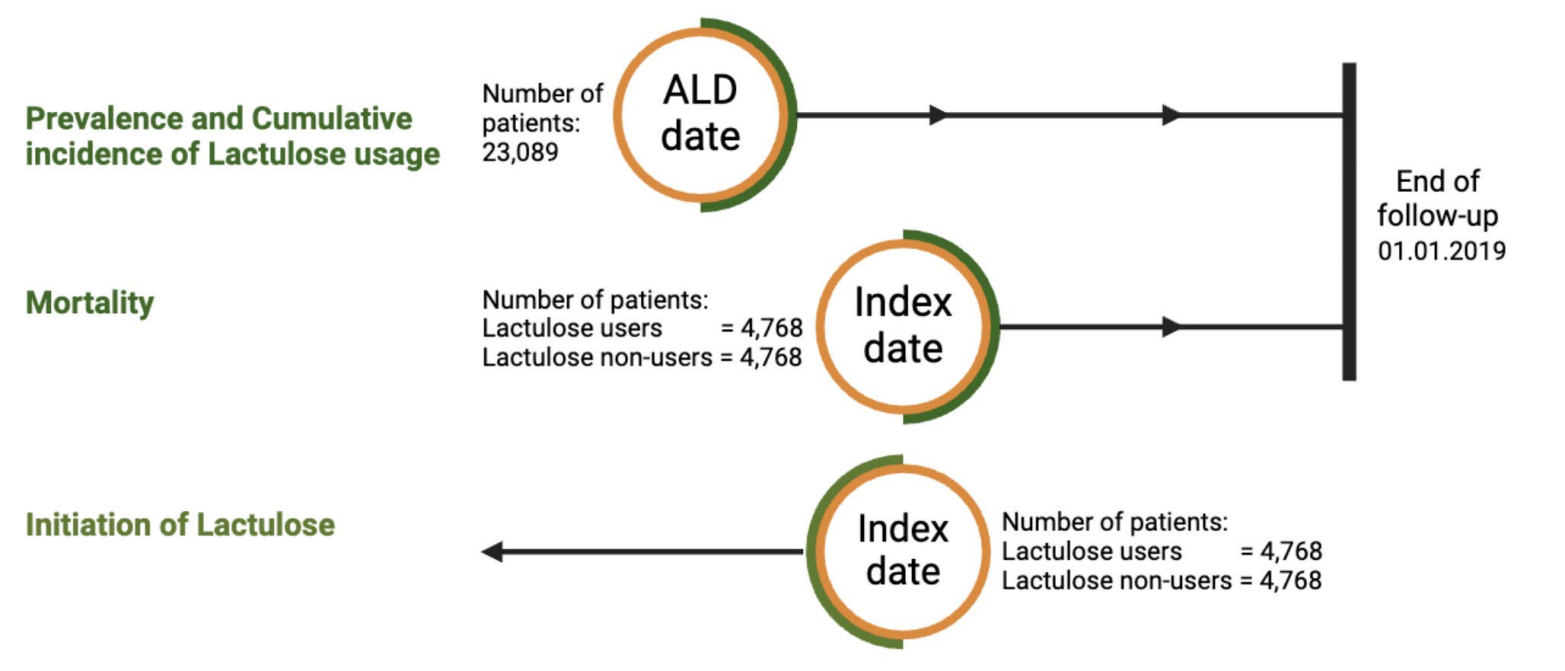
Illustration of the study design

All patients with ALD cirrhosis within our cohort were included to analyze the prevalence of lactulose use and the cumulative incidence of lactulose use from the ALD date. The ALD date represents the first possible date after ALD diagnosis on which patients were able to fill a prescription. (Fig. 1).

Figure 1 Prevalence and cumulative incidence of lactulose use: All patients with a diagnosis of ALD cirrhosis from 1 January 2000 to 1 January 2019 were followed from the date patients enters the cohort (ALD date) until end of follow-up to study prevalence and cumulative incidence of first-time lactulose use after ALD cirrhosis diagnosis. We excluded patients who had used lactulose before their ALD date. The prevalence at time *t *is the proportion of lactulose users at time *t *among those remaining in the cohort at time *t*. The cumulative incidence at time *t *is the proportion of patients who have initiated lactulose use up to time *t *among those who were in the cohort at the beginning of follow-up. Mortality: Cases and matched controls were followed from index date until death, censoring or end of follow-up. Patients who emigrated from Denmark were censored on the emigration date and were not allowed to re-enter the cohort if they later immigrated back to Denmark.

#### Initiation of lactulose in patients with ALD cirrhosis

In this analysis we used a nested case-control design, i.e., a case-control study was nested within our cohort of patients with ALD cirrhosis. The purpose of this design was to match cases (patients who initiate lactulose) and controls (patients who have not [yet] initiated lactulose) on time since cirrhosis diagnosis, which is a very strong predictor of mortality(Jepsen et al. 2010). With this study design, all cohort members are followed from the date of their ALD diagnosis, and time is expressed as ‘time since cirrhosis diagnosis’. When a member fills his or her first prescription for lactulose, he or she is identified as a ‘case’ (labeled “case #1”), and one other cohort member is selected at random as a ‘control’ (labeled “control #1”). The patient labeled “case #1” is removed from the cohort because he/she is no longer at risk of initiating lactulose, and all the others are followed onwards. Later, another patient fills his or her first prescription for lactulose and becomes “case #2”, and simultaneously “control #2” is drawn at random from the cohort. Then “case #2” is removed from the cohort, and the others are followed onwards, and so on until there are no more cohort members at risk of initiating lactulose (or follow-up ends, which happened for our cohort on 1 January 2019) (Fig. 1). After that, we have equal-sized groups of cases and controls and multiple ‘risk sets’, each of them consisting of two patients with ALD matched on time since cirrhosis diagnosis (case #1 and control #1 make up one risk set, etc.). The two members of a risk set share the same “index date”, which is the date on which the set’s case filled his or her first prescription for lactulose. The sampling procedure described here is called ‘risk set sampling’, also known as ‘incidence density sampling’ (Langholz and Clayton 1994). It is possible for a patient with ALD to be selected as a control in multiple risk sets, and it is also possible that a patient with ALD was not in contact with the healthcare system between their date of ALD diagnosis and their index date.

#### Mortality

This analysis included all lactulose users and their matched controls, in whom we analyzed the impact of lactulose usage on mortality from the index date.

### Index date variables

Patient information on the index date was based on data from the National Prescription Registry and the National Patient Registry. It included diagnoses of chronic obstructive pulmonary disease (COPD), diabetes, cardiovascular disease (CVD), severe liver disease (defined as having a diagnosis code for one or more of the following: ascites, portal hypertension, spontaneous bacterial peritonitis or gastrointestinal bleeding) (Jepsen et al. 2008), HCC, and other cancers (Jepsen et al. 2008).

The date defining COPD was the earliest hospital diagnosis code of COPD. The date defining diabetes was the earliest of the following: a hospital diagnosis code of any type of diabetes or prescription of antidiabetic medicine. The date defining CVD was the earliest of the following: a hospital diagnosis code of arterial hypertension, atherosclerosis, or ischemic heart disease. The date defining severe liver disease was the earliest of the following: a hospital diagnosis code of either ascites, portal hypertension, hepatorenal syndrome, spontaneous bacterial peritonitis or gastoesophageal varices with or without bleeding (McPherson et al. 2016). The date defining HCC was the earliest hospital diagnosis code of HCC. The date defining other cancer was the earliest hospital diagnosis code of any type of cancer except HCC. All codes are listed in Supplementary Table 1.

### Statistical analysis

#### Prevalence of lactulose use

The prevalence of lactulose use was computed for each day between the ALD date and end of follow-up as the number of lactulose users in the ALD cirrhosis cohort on a given day divided by the total number of patients in the ALD cirrhosis cohort on that same day.

#### Cumulative incidence of lactulose use

The cumulative incidence of lactulose use was computed for each day between the ALD date and end of follow-up using the cumulative incidence function with death as a competing risk.

#### Initiation of lactulose use in patients with ALD cirrhosis

We used conditional logistic regression to study the association between sex, age and the defined index date variables and the incidence rate ratio of initiating lactulose treatment.

#### Mortality

We used Cox regression to examine the association between initiating lactulose and all-cause mortality, adjusting for sex, age and the above-mentioned index date variables. Cases and controls were followed from their index date until death or until censorship at the end of follow-up, 1 January 2019, whichever came first.

## Results

**Fig. 2:**
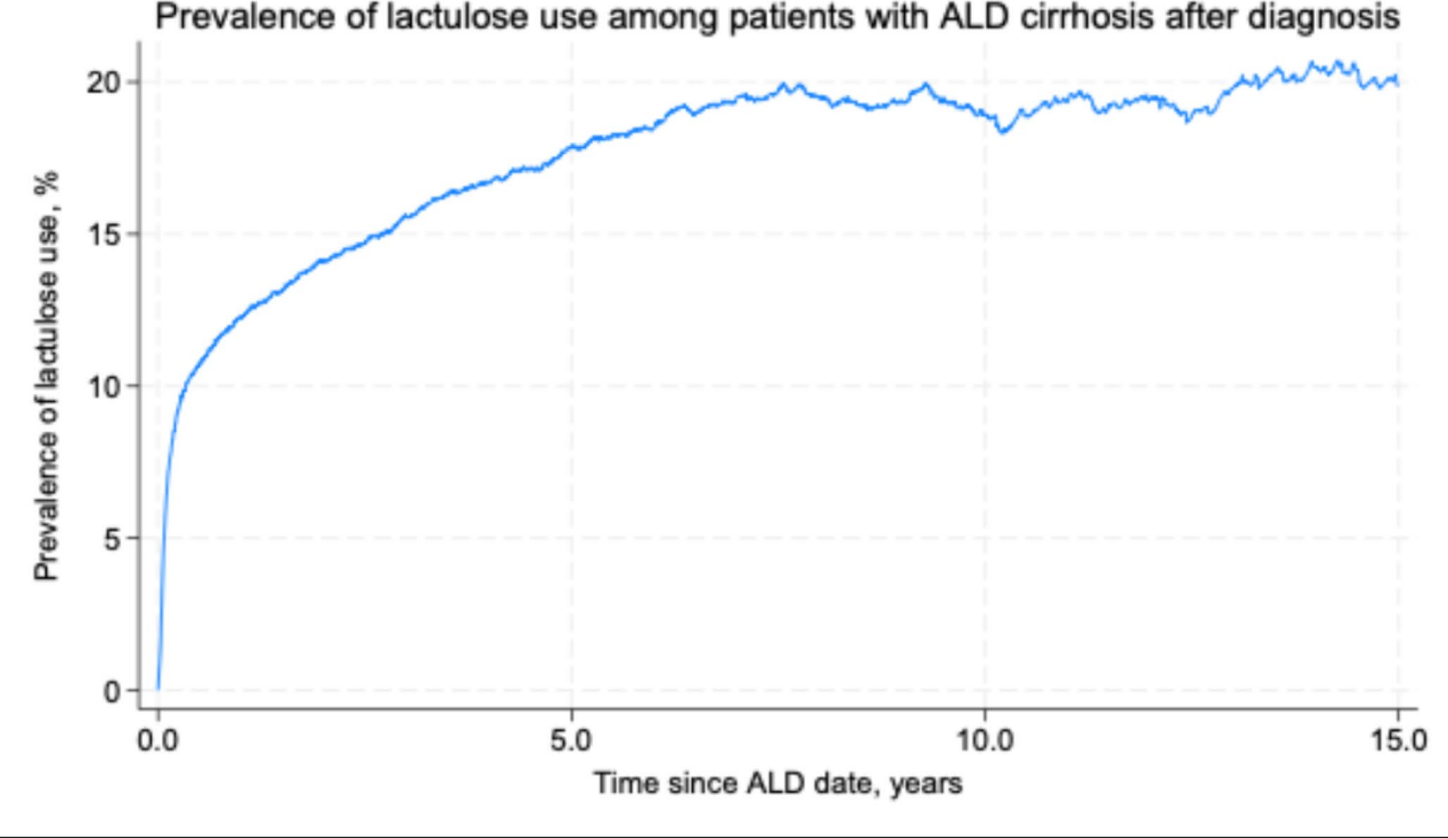
Prevalence of lactulose use among patients with alcohol-related cirrhosis (ALD cirrhosis) with no history of lactulose usage before diagnosis of ALD cirrhosis, according to time since diagnosis of ALD cirrhosis (‘ALD date’)

Our ALD cirrhosis cohort included 23,089 patients. During 30,809 person-years of follow-up, 4,768 patients filled a prescription for lactulose. The prevalence of lactulose usage rose to 11% within the first 6 months, after which it reached 13% after 1 year and 19% after 5 years (Fig. 2).

**Fig. 3:**
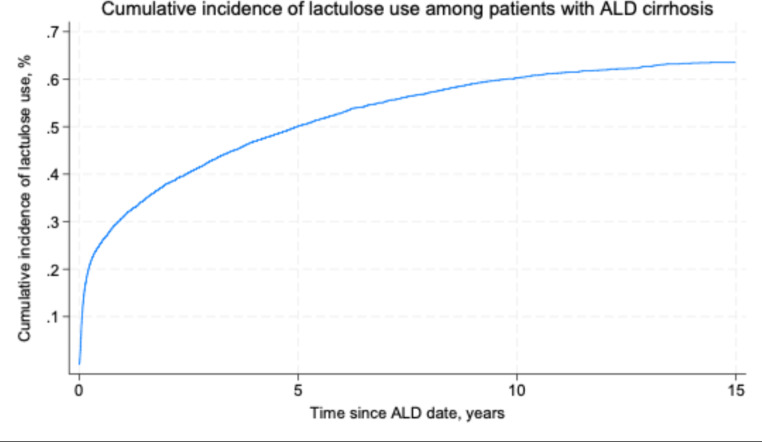
Cumulative incidence of lactulose use among patients with alcohol-related cirrhosis (ALD cirrhosis) with no history of lactulose usage before ALD cirrhosis diagnosis, according to time since diagnosis of ALD cirrhosis (‘ALD date’)

Among our patients with ALD cirrhosis, the 1-year cumulative incidence of lactulose use was 31%, and the 5-year cumulative incidence was 50% (Fig. 3).

During the follow-up 6,053 patients died, yielding a mortality rate of 197 per 1000 person-years (95% CI 192–201) (Table 1).

**Table 1:** Characteristics of patients with alcohol-related cirrhosis have filled or filled their first prescription for lactulose

	Usage of Lactulose		
	Yes	No	Total
No. of patients	4,768	4,768	9,536
Age, median (IQR)	58 (51–64)	57 (50–64)	57 (51–64)
Sex, male	68%	67%	67%
Diabetes	21%	18%	19%
Chronic obstructive lung disease	13%	11%	12%
Cardiovascular disease	30%	27%	28%
Severe liver disease	72%	57%	64%
Hepatocellular carcinoma	4%	2%	3%
Cancer, others	13%	11%	12%
Time at risk	12,613 years	18,196 years	30,809 years

The 4,768 patients who filled a prescription for lactulose were matched with 4,768 controls who had no history of lactulose usage. History of severe liver disease (adjusted odds ratio (OR) = 2.00 [95% confidence interval (95% CI) 1.83–2.18]) and HCC (adjusted OR = 1.73 [95% CI 1.32–2.27]) were strong predictors of initiating lactulose use. Other predictors were older age, history of diabetes, COPD, and non-HCC cancer (Table 2).

**Table 2:** Predictors of initiating lactulose among patients with ALD cirrhosis

	Crude OR [95% CI]	Adjusted OR [95% CI]
Sex, male	1.08 [1.00–1.18]	1.05 [0.96–1.15]
Age, per 10 years	1.12 [1.08–1.17]	1.11 [1.06–1.16]
Diabetes	1.23 [1.11–1.36]	1.12 [1.01–1.25]
Cardiovascular disease	1.17 [1.07–1.28]	1.09 [0.99–1.20]
Chronic obstructive pulmonary disease	1.22 [1.08–1.38]	1.22 [1.07–1.38]
Severe liver disease	1.97 [1.81–2.16]	2.00 [1.83–2.18]
Hepatocellular carcinoma	2.04 [1.57–2.64]	1.73 [1.32–2.27]
Cancer, others	1.23 [1.09–1.40	1.13 [0.99–1.29]

Mortality was higher in lactulose users (71% of lactulose users and 57% of nonusers died during the follow-up period, adjusted hazard ratio (HR) 1.61 [95% CI 1.53; 1.69]). Only HCC was a stronger predictor of mortality than lactulose use (Table 3).

**Table 3:** Hazard ratios for mortality in patients with ALD cirrhosis

	Crude HR [95% CI]	Adjusted HR [95% CI]
Mortality, lactulose versus non-use	1.69 [1.60; 1.78]	1.61 [1.53; 1.69]
Sex, male	1.22 [1.15; 1.28]	1.20 [1.13; 1.27]
Age, per 10 years	1.27 [1.24; 1.31]	1.23 [1.19; 1.26]
Diabetes	1.25 [1.17; 1.33]	1.09 [1.02; 1.16]
Cardiovascular disease	1.22 [1.16; 1.30]	1.03 [0.98; 1.10]
Chronic obstructive pulmonary disease	1.31 [1.22; 1.42]	1.22 [1.13; 1.32]
Severe liver disease	1.25 [1.19; 1.32]	1.17 [1.11; 1.24]
Hepatocellular carcinoma	3.20 [2.79; 3.66]	2.46 [2.14; 2.83]
Cancer, others	1.55 [1.44; 1.67]	1.32 [1.22; 1.43]

## Discussion

We found that the prevalence of lactulose use after diagnosis of ALD cirrhosis rose rapidly to 11% and then climbed slowly to reach 19% within five years after diagnosis of ALD cirrhosis; that the cumulative risk of lactulose use was 31% 1 year after ALD cirrhosis diagnosis; that the presence of cirrhosis complications predicted a high probability of initiating lactulose usage; and that lactulose usage was associated with marked increase in mortality.

Lactulose is in itself a safe medication without severe side effects and usage of lactulose should not result in higher mortality (Luo et al. 2011). Our study supported lactulose as a surrogate marker of HE, since severe liver disease was the strongest predictor of lactulose usage, and lactulose was associated with higher mortality. Other studies have found higher HRs of mortality associated with HE (HRs of, respectively, 4.9 and 2.76 (Patidar et al. 2014; Hanai et al. 2019), which is higher than what was found in our study for lactulose and mortality. Likely reasons for this discrepancy are that lactulose reduces the mortality associated with HE (Tapper and Parikh 2023), and that some lactulose users did in fact not have HE (Ruszkowski and Witkowski 2019). We conclude that lactulose does not reflect the full effect of HE in ALD cirrhosis patients, but it may still be useful as a simple tool for identifying patients with HE in larger cohorts.

We used patient information based on data from the Danish National Prescription Registry and the National Patient Registry to create a population-based cohort of patients with ALD cirrhosis, resulting in a total study population of 9,536 patients from a cohort of 23,000 patients diagnosed with ALD cirrhosis from 2000 till 2019. This ensured a large sample size with great statistical power and limited the influence of selection bias (Thygesen and Ersbøll 2014).

It was a limitation of our study that we could not determine the indication for starting lactulose. Besides treatment of HE, lactulose is a commonly used laxative, and the indication for prescribing a drug is not recorded in the registry, and we did not have access to patients’ medical records. We excluded patients with a history of lactulose usage before they were diagnosed with ALD cirrhosis, assuming that those patients had likely used lactulose to treat constipation. With this exclusion criterion, our findings align with previous studies: The prevalence of lactulose use we found was compatible with the prevalence of HE in patients with ALD cirrhosis at diagnosis (11%) in a Danish population-based cohort study based on data from patient records (Jepsen et al. 2010). The cumulative risk of lactulose use among patients with ALD cirrhosis was 31% 1 year after ALD cirrhosis diagnosis, and this estimate is comparable to the 1-year cumulative risk of up to 25% found in a prospective study from the United States (Tapper and Parikh 2023). Therefore, we believe that lactulose was most likely prescribed to treat HE in our study.

The possibility of buying lactulose over-the-counter was an additional limitation of our study that may have led us to underestimate the prevalence of lactulose use. However, in our clinical experience most patients with ALD cirrhosis have lactulose prescribed and not merely recommended to them, as the prescription serves as a reminder to the patient and his or her general practitioner. We believe that virtually all lactulose-using patients with ALD cirrhosis have filled a prescription for lactulose at some point, hence any underestimation of lactulose use due to over-the-counter sales is negligible.

The findings of our study must be evaluated in the context of the study design, and the validity of ICD-10 diagnosis codes for ALD cirrhosis and comorbidities and the validity of the National Presciption Registry were crucial. Previous studies have indicated that the positive predictive value (PPV) of diagnosis codes for ALD cirrhosis is at least 80% (Vestberg et al. 1997; Jepsen et al. 2010; Dam Fialla et al. 2012) and that the PPV of using medication codes to identify patients with HE is 77% (Tapper et al. 2021). The diagnosis codes used to define comorbidities in our study vary in validity, but have a PPV of at least 67% (Schmidt et al. 2015). We did not have access to measurements of blood pressure, and diagnosis codes of hypertension often indicate worsening rather than onset of hypertension. Therefore, we used the variable ‘cardiovascular disease’, which was a composite variable including arterial hypertension, atherosclerosis, or ischemic heart disease. False-positive diagnosis codes for ALD cirrhosis may have caused us to overestimate the true association between lactulose use and mortality (because users of lactulose were more likely than nonusers to truly have ALD cirrhosis), but we believe that there were too few false-positive diagnoses to materially affect our conclusions. Moreover, even our nonusers of lactulose had a high mortality, suggesting that they did have ALD cirrhosis. In the National Presciption Registry only information about drugs dispensed and purchased by patients is recorded (Pottegård et al. 2017), so primary non-compliance is not an issue (Beardon et al. 1993; Furu et al. 2010). We cannot be certain that patients who filled a prescription for lactulose truly used lactulose, though, so we may have overestimated the true prevalence of lactulose use.

Another noticeable limitation of our study was that we did not include data on rifaximin. Rifaximin is the standard second-line treatment of HE, but in Denmark rifaximin is handed out at the hospitals to the patients due to costs, which is not being documented at an individual level and therefore we could not collect data on prescriptions of rifaximin.

## Conclusion

Our study shows that lactulose is an often-used medication used to treat patients with severe liver disease. First-time usage of prescribed lactulose is associated with higher mortality in patients with ALD cirrhosis even though lactulose itself is a relatively harmless medication, and the prevalence of lactulose use reflects the prevalence of HE. We conclude that lactulose use can be used to estimate the prevalence of HE, and that it can be used as a surrogate for HE to reduce confounding. We would not use lactulose use as a surrogate for HE in a study with HE as an outcome; that would require stronger evidence and more detailed data than what we had available in this registry-based study. Even so, our findings may be important for epidemiological studies of HE in patients with cirrhosis.

## Electronic supplementary material

Below is the link to the electronic supplementary material.

Supplementary Material 1

## Data Availability

According to Danish law, we can only access the data through a secure connection to a remote server hosted by the Danish Health Data Authority. We cannot download the data, let alone share it. We will perform additional analyses and share our code upon reasonable request.
