# Supplementary material for: Lactulose use among patients with alcohol-related liver cirrhosis as a surrogate marker of hepatic encephalopathy: prevalence and association with mortality - a Danish nationwide cohort study
Source: Metab Brain Dis. 2025 Jan 18;40(1):107. doi: 10.1007/s11011-025-01533-w (PMC11742859; doi:10.1007/s11011-025-01533-w)
Supplement: Supplementary file 1 — Supplementary Material 1 [file 11011_2025_1533_MOESM1_ESM.docx]

**Supplementary tables**

**Title:** Lactulose use among patients with alcohol-related liver cirrhosis as a marker of hepatic encephalopathy: prevalence and association with mortality - a Danish nationwide cohort study

Journal: Metabolic Brain Disease

**Authors:** Emma Celia Herting^1,2^, [ec.herting@gmail.com](mailto:ec.herting@gmail.com),;Morten Daniel Jensen^1,2^; Peter Jepsen^1,2,3^,

**Institutional affiliations:** ^1^Department of Hepatology and Gastroenterology, Aarhus University Hospital, Aarhus, Denmark**,** ^2^Institute of Clinical Medicine, Aarhus University, Aarhus, Denmark**,** ^3^Department of Clinical Epidemiology, Aarhus University Hospital, Aarhus, Denmark

| Supplementary table 1 | Codes used to identify patient characteristics and events | |
| --- | --- | --- |
|  | **ICD-10** | **ATC** |
| **Alcohol related liver cirrhosis** | K70.3x, DK70.4x |  |
| **Lactulose** |  | A06AD11 |
| **Diabetes** | E10.x, E11.x, E12.x, E13.x, E14.x |  |
| Antidiabetic |  | A10 |
| **Cardiovascular diseases** |  |  |
| Hypertension | I10.x-I15.x |  |
| Atherosclerosis | I70.x, I67.2, K55.1A |  |
| Ischemic heart disease | I20.x-I25.x |  |
| **Chronic obstructive pulmonary disease** | J44.x |  |
| **Severe liver disease** |  |  |
| Portal hypertension | K766.x |  |
| Ascites | R18.x |  |
| Gastrointestinal bleeding | I85.x, K92.x |  |
| Hepatorenal syndrome | K76.7 |  |
| Spontaneous bacterial peritonitis | K65.8I |  |
| Liver insufficiency | K72.x |  |
| **Hepatocellular carcinoma** | C22.0 |  |
| **Cancer, other** | Cx (except C22.0) |  |
